# Single Port 11 mm Laparoscopic Hysterectomy Performed on a 32-Year-Old Female with Adhesive Disease

**DOI:** 10.1155/2020/3757391

**Published:** 2020-06-26

**Authors:** Greg J. Marchand, Ali Azadi, Sienna Anderson, Stacy Ruther, Sophia Hopewell, Giovanna Brazil, Katelyn Sainz, Hannah Wolf, Alexa King, Janelle Vallejo, Kelly Ware, Kaitlynne Cieminski, Anthony Galitsky, Akarshi Kaue Brar

**Affiliations:** ^1^The Marchand Institute for Minimally Invasive Surgery, Mesa, AZ, USA; ^2^Star Urogynecology, Peoria, AZ, USA; ^3^Washington University of Health and Science, San Pedro, Belize; ^4^International University of Health Sciences, Basseterre, Saint Kitts and Nevis

## Abstract

In the field of minimally invasive surgery, there is constant drive to devise and execute the most minimally invasive surgeries possible. By the very nature of laparoscopy and robotic surgery, what one can accomplish with several ports of a given size will invariably be studied and attempted with fewer ports and with ports of smaller sizes. Although more complex pathology may require a more invasive approach, surgical cases without serious complicating factors may be amenable to extremely minimally invasive procedures. We report one such case where a 32-year-old female suffering from adenomyosis and endometriosis was able to receive a laparoscopic single-port hysterectomy and bilateral salpingo-oophorectomy through a single 11 mm port created with a blunt trochar.

## 1. Introduction

Unlike other specialties which are defined by the general field of medicine they pertain to, “minimally invasive surgery” itself can be understood as a challenge to its practitioners; its very name encouraging them to pursue a more minimally invasive approach. The specific issue we sought to address here was attempting the most minimally invasive single-port hysterectomy ever performed, while still performing meaningful laparoscopic visualization of the abdomen and the realistic expectation to be able to realistically operate in the abdomen from a laparoscopic approach. This meant that we specifically did not wish to perform a procedure that one could be considered a laparoscopy then followed by vaginal hysterectomy, and desired meaningful laparoscopic access to deal with issues such as adhesions, mobilization of the bladder flap, or performing a bilateral salpingo-oophorectomy without significant vaginal assistance. After researching the literature, we were not able to find any single-port hysterectomies performed through a port size of smaller than 15 millimeters [1]. The authors also wished to exclude cases with no significant pathology or adhesive disease, as the purpose of describing the technique is to show that the technique can be used in many challenging situation, not to demonstrate that the technique can be successful on the easiest of hysterectomies. All authors strongly contend that hysterectomies that can be performed through a completely vaginal technique should be, and that a vaginal hysterectomy, (or zero port hysterectomy), is superior to a laparoscopic hysterectomy if it can be accomplished [2].

Multiple authors have documented the feasibility of single-incision laparoscopic hysterectomy [3]. Many authors have commented that the idea, although novel, does not significantly improve intraoperative pain, recovery, or surgical cosmesis [4]. The most commonly used system is a robot-assisted single-port system. All systems, to the knowledge of the authors, require incisions greater than 15 mm in the umbilicus [5, 6]. We examined different single-port systems and combined available instrumentation to create a feasible, repeatable technique for performing a laparoscopic single-site hysterectomy using only an 11 mm umbilical incision that is created with a blunt laparoscopic trochar.

## 2. Case Report

A 32-year-old woman with endometriosis, adenomyosis, and chronic pelvic pain with recurrent ovarian cysts presented for laparoscopic hysterectomy with bilateral salpingo-oophorectomy. Patient had previously tried more conservative surgeries and medical treatments including a 6-month course of leuprolide acetate and multiple surgeries for fulguration of endometriosis. The patient completed her desired childbearing and requested definite treatment. The patient had a history of prior bilateral salpingectomy and one prior cesarean section. The patient had confirmed endometriosis at previous laparoscopic exploration and was suspected to suffer from adenomyosis based on cyclic pain and pain that seemed to originate from the uterus with gentle palpation with the vaginal ultrasound probe. Patient was extensively counseled to the risks of bilateral salpingo-oophorectomy and offered more conservative surgical options including hysterectomy without bilateral salpingo-oophorectomy. The patient refused more conservative treatments, citing her fear of the necessity of future surgeries for endometriosis or ovarian cysts, the desire for definitive treatment of endometriosis, as well her fear of ovarian cancer in the future despite no family history. Patient politely refused BRCA testing citing that it would not influence her decision for bilateral salpingo-oophorectomy. The total operative time was 38 minutes, and the estimated blood loss was 100 cc. The patient was discharged 18 hours after surgery, and the recovery was uneventful. The final pathology report showed endometriosis and adenomyosis.

## 3. Methods

**Figure 1:**
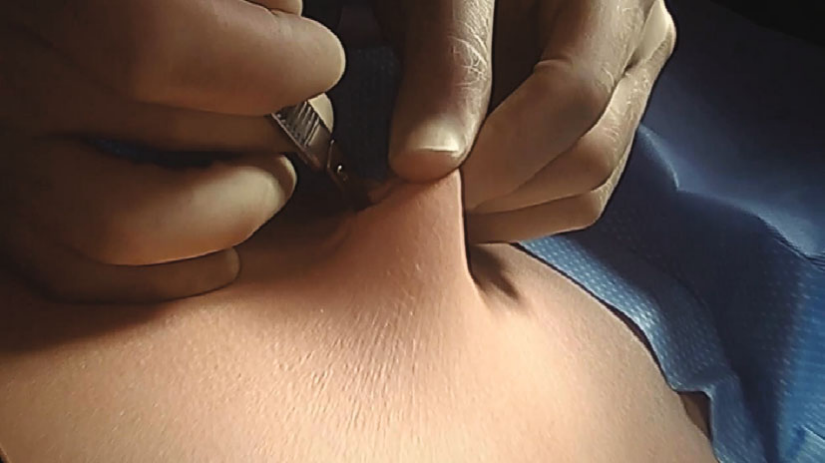
An 11-millimeter incision is made at the bottom of the patient's umbilicus with an 11-blade scalpel.

**Figure 2:**
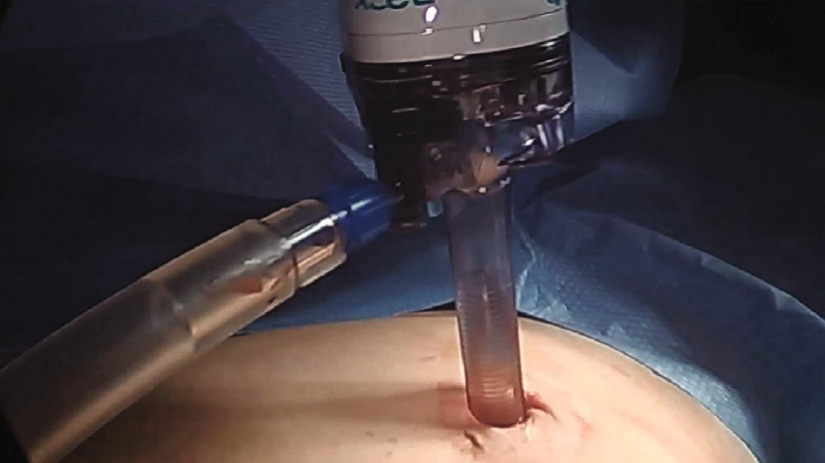
A blunt 11 mm laparoscopic trochar is utilized to make the entry into the abdominal cavity, in order to avoid a sharp dissection into the abdomen which would result in a larger fascial footprint.

**Figure 3:**
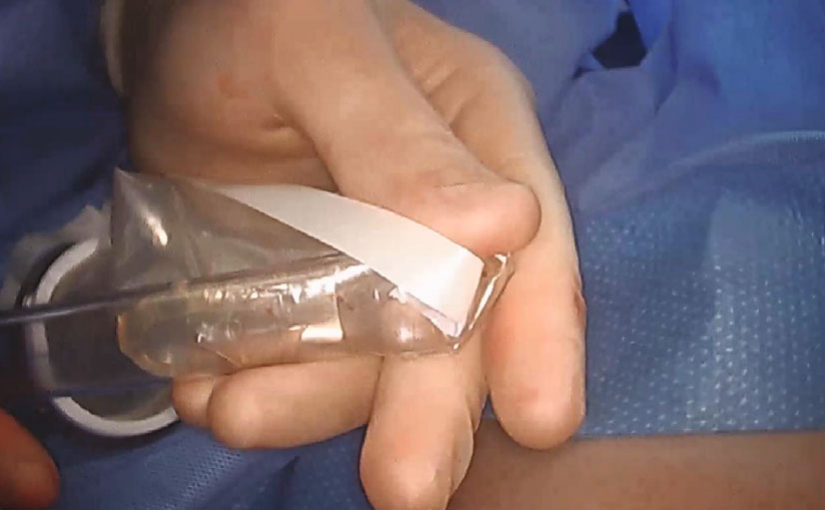
A multiport device is then loaded into the introducer, for insertion into the abdomen.

**Figure 4:**
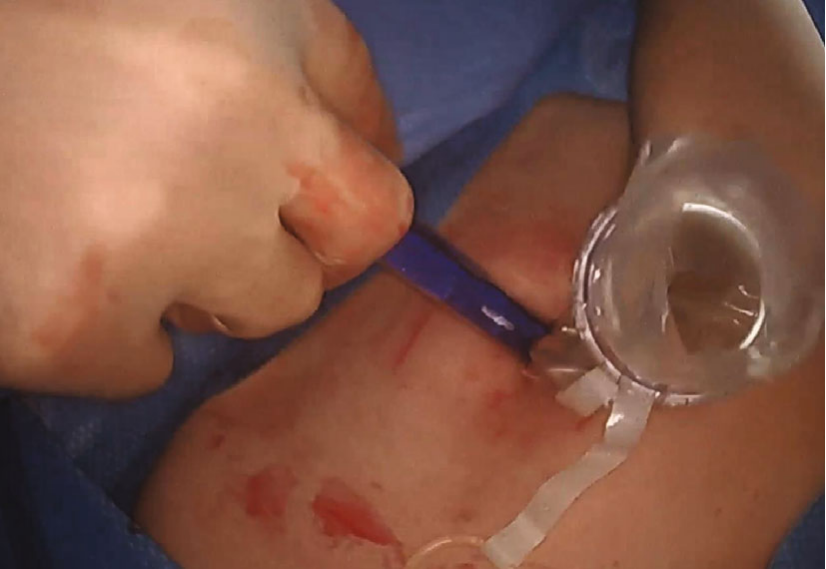
The multiport device is inserted through the abdominal incision after withdrawing the 11 mm blunt trochar. This ensures the incision width will not exceed 11 mm and has been created by blunt (not sharp) entry, which further decreases the change of postoperative hernia.

**Figure 5:**
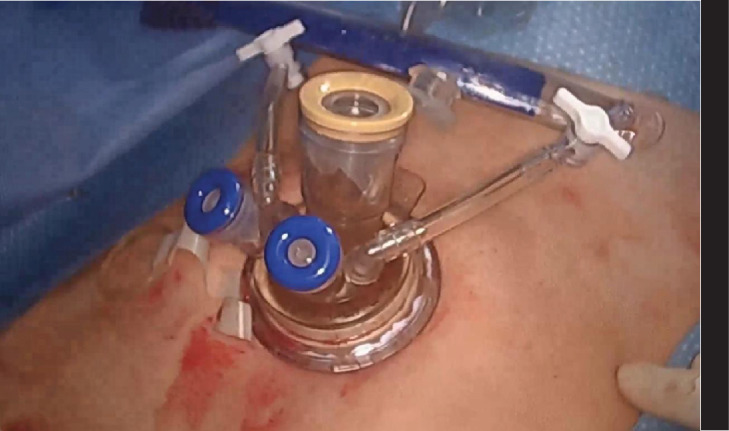
The multiport device is in place, and the laparoscopic hysterectomy can proceed with one or two instruments in addition to the 5 mm laparoscope.

**Figure 6:**
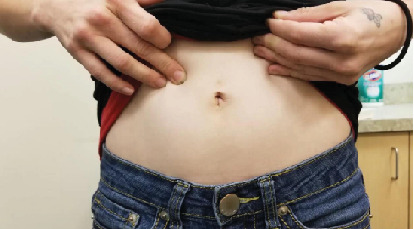
The patient's abdomen at a visit 4 weeks after surgery. No scars are visible.

**Figure 7:**
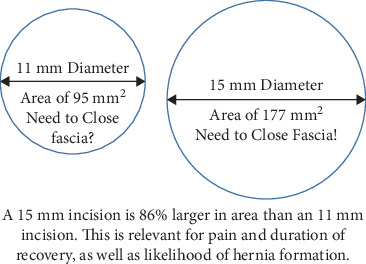
Secondary to the fact that laparoscopic incisions are stretched into a circular shape by the penetrating instrumentation, even a small decrease in the size of a fascial incision will greatly decrease the area of the opening that can pass through that incision. This figure compares the large jump from an area of 95 mm^2^ to 177 mm^2^ when increasing the umbilical incision by only 3 mm.

We devised a technique for laparoscopic single-port hysterectomy based on the concept that a bluntly created incision would be less likely to herniate than a sharply created incision. Therefore, after creating the initial skin incision with an 11-blade scalpel (Figure 1), rather than perform an open dissection that would result in a large incision and a much larger fascial footprint, we then place an 11 mm blunt laparoscopic trochar (Figure 2) into the incision after insufflating with a veress needle. The multiport device is then loaded into its introducer (Figure 3) and is inserted into the abdominal cavity after removing the 11 mm port from the umbilical incision (Figure 4). The multiport device can then be installed and actively utilized to perform the hysterectomy through only an 11 mm incision. This can include inserting one or two five-millimeter-diameter instruments, as well as a five-millimeter laparoscope, to complete the hysterectomy through any method the surgeon is comfortable with (Figure 5). Following this, the uterine pedicles can be divided with a bipolar power coagulation and division device, and the circumferential colpotomy is made with a monopolar cautery set to 30 watts of coagulating current with a laparoscopic hook extender. The vaginal cuff is sewn from the vaginal approach and the ovaries and tubes are removed after removal of the uterus. The patient returned 4 weeks postoperatively and no scars were visible (Figure 6). We believe this technique to be significantly different from any previously described techniques because of the usage of an 11 mm blunt trochar to create the umbilical incision. This creates a reproducible footprint in the fascia that should be identical and reproducible regardless of circumstances. By keeping the incision bluntly created and small, we believe we minimize the risk of postoperative herniation (Figure 7).

## 4. Discussion

In the case of this 32-year-old female, this technique was designed to facilitate the hysterectomy through the smallest incision possible, as well as to perform the technique through a bluntly created incision to further minimize the chance of postoperative herniation. We aimed to perform the procedure using only commercially available materials, although the authors agree that specialized equipment could be manufactured that would allow completion of the procedure through an even smaller incision.

As discussed in Introduction, this technique is considered by the authors to be inferior to a purely vaginal approach. The authors feel that in a patient with no known pathology that would necessitate abdominal surgery, a vaginal technique would be preferable, especially if the ovaries and fallopian tubes were to be retained at time of surgery. In a patient such as this, however, with scar tissue from prior surgery and the expectation of adhesive disease from endometriosis, our technique may be an excellent minimally invasive alternative.

## 5. Conclusion

Our described technique is a feasible, reproducible procedure for hysterectomy and may improve cosmesis and postoperative pain over traditional laparoscopic and single-port techniques.

## Data Availability

All data discussed is presented in the text of the case report.
